# A Machine Learning Approach to Assess Patients with Deep Neck Infection Progression to Descending Mediastinitis: Preliminary Results

**DOI:** 10.3390/diagnostics13172736

**Published:** 2023-08-23

**Authors:** Shih-Lung Chen, Shy-Chyi Chin, Kai-Chieh Chan, Chia-Ying Ho

**Affiliations:** 1 Department of Otorhinolaryngology & Head and Neck Surgery, Chang Gung Memorial Hospital, New Taipei City 333, Taiwan; 2 School of Medicine, Chang Gung University, Taoyuan 333, Taiwan; 3 Department of Medical Imaging and Intervention, Chang Gung Memorial Hospital, New Taipei City 333, Taiwan; 4 Division of Chinese Internal Medicine, Center for Traditional Chinese Medicine, Chang Gung Memorial Hospital, Taoyuan 333, Taiwan

**Keywords:** artificial intelligence, deep neck infection, descending mediastinitis, machine learning

## Abstract

Background: Deep neck infection (DNI) is a serious infectious disease, and descending mediastinitis is a fatal infection of the mediastinum. However, no study has applied artificial intelligence to assess progression to descending mediastinitis in DNI patients. Thus, we developed a model to assess the possible progression of DNI to descending mediastinitis. Methods: Between August 2017 and December 2022, 380 patients with DNI were enrolled; 75% of patients (*n* = 285) were assigned to the training group for validation, whereas the remaining 25% (*n* = 95) were assigned to the test group to determine the accuracy. The patients’ clinical and computed tomography (CT) parameters were analyzed via the *k*-nearest neighbor method. The predicted and actual progression of DNI patients to descending mediastinitis were compared. Results: In the training and test groups, there was no statistical significance (all *p* > 0.05) noted at clinical variables (age, gender, chief complaint period, white blood cells, C-reactive protein, diabetes mellitus, and blood sugar), deep neck space (parapharyngeal, submandibular, retropharyngeal, and multiple spaces involved, ≥3), tracheostomy performance, imaging parameters (maximum diameter of abscess and nearest distance from abscess to level of sternum notch), or progression to mediastinitis. The model had a predictive accuracy of 82.11% (78/95 patients), with sensitivity and specificity of 41.67% and 87.95%, respectively. Conclusions: Our model can assess the progression of DNI to descending mediastinitis depending on clinical and imaging parameters. It can be used to identify DNI patients who will benefit from prompt treatment.

## 1. Introduction

Deep neck infection (DNI) is a fatal bacterial infectious disease that affects the deep cervical spaces [1,2]. It can give rise to airway compromise, making it critical to secure patients’ airways [3]. DNI can cause other complications, depending on the severity of the infection and the direction of invasion. Descending mediastinitis, one of complications of DNI, is a fatal infection of the mediastinum. This illness occurs when DNI spreads along the deep cervical spaces and progresses to the mediastinum [4,5,6]. Even though it is not prevalent, it can cause sepsis and poor outcomes [7]. The management of descending mediastinitis is challenging, and multidisciplinary cooperation is recommended as delayed diagnosis and incomplete surgical drainage can lead to mortality [8]. Due to the variation in causes and locations of the infection, no standard treatment protocol has been established for descending mediastinitis [9].

Artificial Intelligence (AI) technology has been developed for more than half a century. AI enables machines to perform works that basically require human intellect and cognitive performance [10]. Machine learning is a science of AI, and it has been growing in medical applications [11]. Machine learning can be applied to aid decision support and improve clinical practice [12]. To develop predictive models, usually depending on structured data, machine learning models can benefit from the leverage of information from large data sets to strengthen the model [13]. The *k*-NN method is a kind of machine learning that can be used for classification. Other methods such as eXtreme Gradient Boosting (XGboost), t-Distributed Stochastic Neighbor Embedding (t-SNE), and Principal Component Analysis (PCA) are also effective algorithms for machine learning [14,15,16].

However, even with such advanced AI tools, is currently no available model to evaluate the risk of progression of DNI to descending mediastinitis. If this model is feasible, such a tool could identify patients at risk for mediastinitis and enable early treatment. Therefore, we attempt to use a dataset from our hospital and aim to develop a model for predicting the progression of DNI to descending mediastinitis in this research.

## 2. Materials and Methods

This retrospective study enrolled 380 DNI patients who were admitted to the Chang Gung Memorial Hospital, Linkou main branch, Taiwan, between August 2017 and December 2022. A diagnostic computed tomography (CT) scan was performed on the patients. The empirical antibiotics used were ceftriaxone (1 g, q12h) as well as metronidazole (500 mg, q8h) [17]. The antibiotic regimes were modified depending on the final pathogen cultures.

### 2.1. CT Measurements

**Figure 1:**
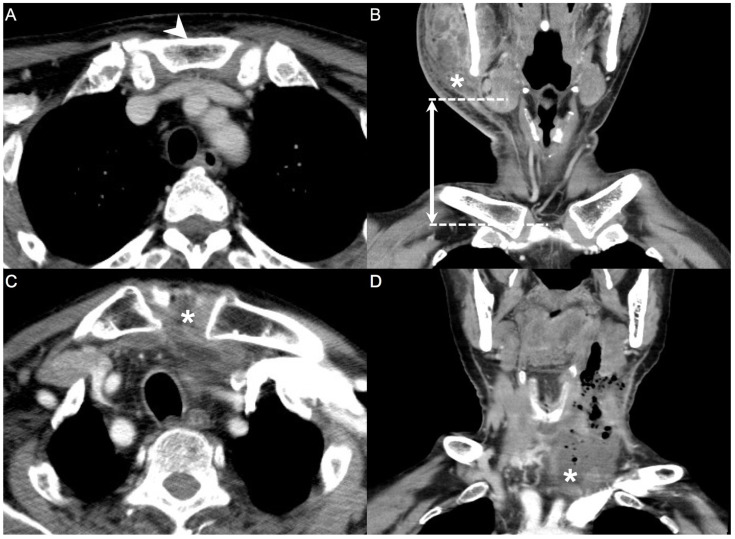
Computed tomography (CT) parameters. Distance measured from the level of the abscess to the level of the sternal notch (**A**,**B**). Downward invasion of the abscess to the sternal notch, leading to mediastinitis (**C**,**D**). Arrowhead: sternal notch; asterisk (*): deep neck abscess; dotted line: horizontal level; double arrow: distance between the level of the abscess and the level of the sternal notch in CT (300 × 300 dpi).

Imaging is fundamental to confirm the diagnosis of DNIs, and a CT scan is the most utilized tool. A CT scan can determine the source of infection, identify the extent of DNI invasion, and provide early recognition of complications [18]. As parameters for the model in this study, the maximum diameter of the abscess in the axial, coronal, and sagittal planes was measured in a CT scan. The nearest vertical distance from the abscess to the level of the sternal notch in the coronal and sagittal planes was also measured. These measurement results were input as the radiological parameters in the model (Figure 1).

### 2.2. Data Collection

We recorded essential clinical variables to establish the model for predicting the progression to mediastinitis (Table 1). These clinical variables, as well as the aforementioned maximum diameter of the abscess and shortest distance from the abscess to the level of the sternal notch, were entered into the model. The progression and non-progression were labeled by at least one otolaryngologist (S.L.C.) and one radiologist (S.C.C.). Finally, we evaluated and compared whether the patients in the training and test groups progressed to mediastinitis.

### 2.3. k-Nearest Neighbor (k-NN) Method

**Figure 2:**
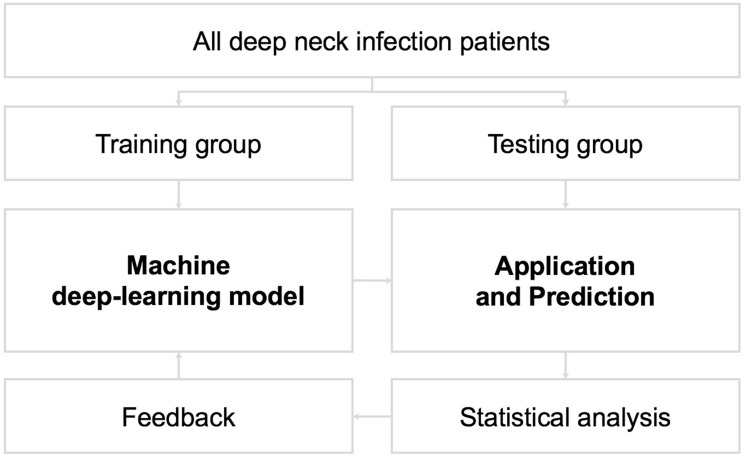
Algorithm of training and test datasets for the model. The feedback arrows in the figure are intended to represent future continuous learning of the system, but those are not directly applicable to the reported results in the current study (300 × 300 dpi).

**Figure 3:**
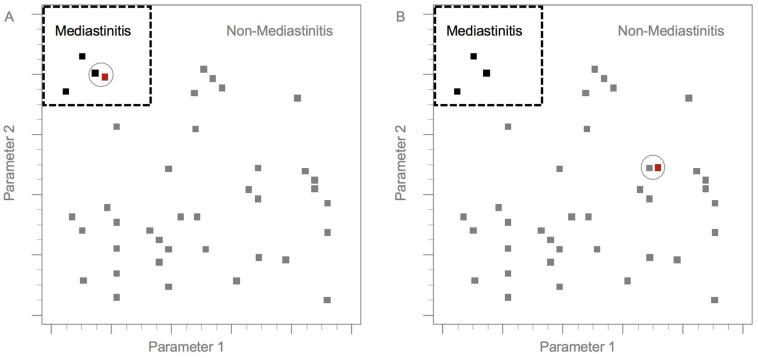
Diagram of the *k*-nearest neighbor model. In (**A**,**B**), black squares represent training dataset patients who had progressed to mediastinitis. Grey squares represent training dataset patients who did not progress to mediastinitis. Red squares represent test group patients. The dotted line divides patients into those who did and did not progress to mediastinitis. Circles represent the nearest neighbors to the test and training group instances (300 × 300 dpi).

To establish a practical model, the dataset was divided into training and test groups [19,20]. The model was validated via the training group, whereas the model’s performance was evaluated in a formerly unseen test group [10]. In general, 75% of the data (*n* = 285) were randomly assigned to the training group, and the remaining 25% (*n* = 95) were distributed to the test group (Figure 2). In this research, we applied the *k*-NN method as the practical model. The *k*-NN is one of the algorithms for supervised learning in machine learning. The *k*-NN algorithm is used to classify each test instance based on similarity to neighbors in the training instance. We converted continuous and categorical variables into z-scores and subtracted the mean score from the individual scores. Furthermore, the remaining values were divided by the standard deviation [21]. In the *k*-NN method, the Euclidean distance D indicates the distance between two points in the *n*-dimensional space, with each *n*-dimension corresponding to each *n*-feature [22,23,24]. The final classification and output depend on the distances between the test and training data (Figure 3) [20,21,25,26,27,28].

### 2.4. Exclusion Criteria

Patients with cervical necrotizing fasciitis [29], severe cardiopulmonary disease, history of head and neck malignancy [30,31], esophageal perforation, aspiration pneumonia during admission, immunocompromised status, or failure to collect complete variables were excluded.

### 2.5. Ethics Statement 

The requirement for informed consent was waived because the data were collected retrospectively and anonymized before analysis. The present study protocol was approved by the Institutional Review Board of Chang Gung Medical Foundation (approval no. 202300075B0). 

### 2.6. Statistical Analysis

Classification accuracy (mediastinitis vs. non-mediastinitis) was calculated as the ratio between the number of correctly classified patients and the total number of patients [21]. Sensitivity and specificity were calculated. Sensitivity (true positive rate) refers to the proportion of correctly identified positive (mediastinitis) patients, whereas specificity (true negative rate) refers to the proportion of correctly identified negative (non-mediastinitis) patients. Data were analyzed using MedCalc (ver. 18.6; MedCalc, Ostend, Belgium) and Excel (Microsoft Corp., Redmond, WA, USA) [32] software. The Mann–Whitney *U* test was used to analyze continuous variables, and the data were displayed as means and standard deviations. For group differences in category variables, the two-tailed Fisher’s exact test was performed. *p*-values less than 0.05 were considered to indicate a statistical difference.

## 3. Results

Table 1 presents the demographic and clinical data of the study participants. In total, 380 DNI patients with a mean age of 51.12 ± 18.87 years were enrolled, including 255 males (67.11%) and 125 females (32.89%). The mean chief complaint period was 5.06 ± 4.47 days. The mean WBC count, CRP level, and blood sugar levels were 14,908.41 ± 5753.66 µL, 154.68 ± 99.54 mg/L, and 142.56 ± 73.42 mg/dL, respectively. In total, 142 (37.36%) patients had DM. 

The parapharyngeal, submandibular, and retropharyngeal spaces were involved in 182 (47.89%), 167 (43.94%), and 100 (26.31%) patients, respectively. Multiple spaces (≥3) were involved in 124 (32.63%) patients. 

Tracheostomies were performed in 46 (12.11%) patients. In CT, the mean maximum diameter of the abscess was 6.23 ± 2.96 cm, and the shortest distance from the abscess to the level of the sternal notch was 6.11 ± 3.92 cm. Progression to mediastinitis was observed in 30 (7.89%) DNI patients.

Table 2 compares the patients in the training (*n* = 285) and test (*n* = 95) groups. In terms of age (50.75 ± 18.71 vs. 52.26 ± 19.38 years; *p* = 0.521), gender (*p* = 0.378), chief complaint period (5.33 ± 4.89 vs. 4.25 ± 2.71 days; *p* = 0.213), WBC (14,622.45 ± 5695.52 vs. 15,766.31 ± 5871.63 µL; *p* = 0.090), CRP (150.97 ± 98.72 vs. 165.79 ± 101.66 mg/L; *p* = 0.191), blood sugar (140.91 ± 72.55 vs. 147.51 ± 76.15 mg/dL; *p* = 0.090), and DM (*p* = 0.806), there was no statistical significance observed between the groups regarding clinical variables.

In the deep neck space, the parapharyngeal (48.07 vs. 47.36%; *p* = 1.000), submandibular (41.75 vs. 50.52%; *p* = 0.152), retropharyngeal (23.85 vs. 33.68%; *p* = 0.079), and multiple spaces involved (≥3) (31.22 vs. 36.84%; *p* = 0.315), there was no significant difference detected.

For airway protection, there was no difference in tracheostomy performance (*p* = 0.588).

In imaging parameters, there was no statistical difference found on the maximum diameter of the abscess (6.08 ± 2.92 vs. 6.66 ± 3.04 cm; *p* = 0.072) or the nearest distance from the abscess to the level of the sternum notch (6.26 ± 3.74 vs. 5.69 ± 4.41 cm; *p* = 0.210). Furthermore, there was no significant difference noted in progression to mediastinitis (*p* = 0.075).

Our model had an accuracy for prediction of DNI progression to mediastinitis of 82.11% (78/95 patients), with sensitivity and specificity of 41.67% and 87.95%, respectively.

In Figure 1, the measurement of the distance from the level of the abscess to the level of the sternal notch was displayed in the CT scan. It’s the most important imaging parameter in our research. Another important CT parameter is the maximum diameter of the abscess.

The algorithm for training and testing datasets in this research for the model is demonstrated in Figure 2. This figure clearly shows the main structure of this study. However, the feedback arrows are related to the potential future enhancements of the system through continuous learning and are not directly applicable to the reported results in the current study.

Figure 3 displays the main principle of the *k*-nearest neighbor model. The black dots represent training dataset patients who had progressed to mediastinitis, while the gray dots represent training dataset patients who did not progress to mediastinitis. The red dots represent test group patients. The dotted line divides patients into those who did and did not progress to mediastinitis. Circles represent the nearest neighbors to the test and training group instances.

**Figure 4:**
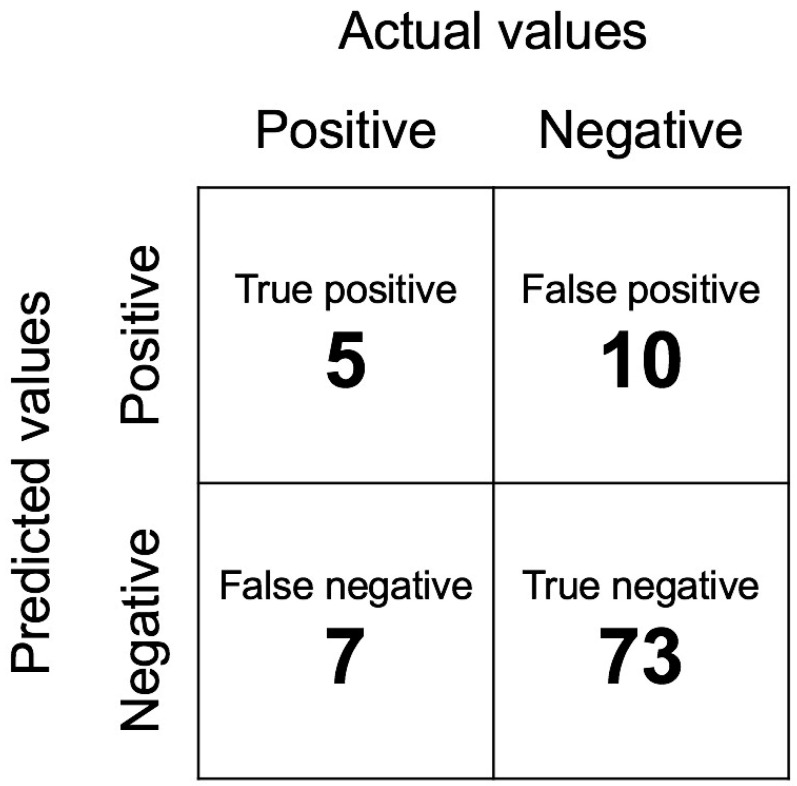
Confusion matrix of the model. The numbers of true positives, false positives, false negatives, and true negatives were 5, 10, 7, and 73, respectively. The F1 score is 0.37 (300 × 300 dpi).

Figure 4 shows the information in the confusion matrix. The numbers of true positives, false positives, false negatives, and true negatives were 5, 10, 7, and 73, respectively. The F1 score is 0.37.

## 4. Discussion

DNIs are highly associated with odontogenic infections as well as poor oral hygiene [33]. The factors connected to the progression of odontogenic infections to DNI are linked to public health, a lack of prevention, and insufficient medical management [34]. The long-term hospitalization caused by DNIs will be a huge burden not only on patients and their families, but also on the entire medical system [35]. Thus, it is essential to identify the clinical, laboratory, radiological, and pathogen factors that predispose DNI patients to life-threatening complications [35,36,37,38,39]. In this era of aging, some authors have found certain clinical differences in DNIs between elderly patients and adult patients [40].

Descending mediastinitis can occur as a complication of DNI. This rare but fatal illness involves a severe soft tissue infection that progresses along the fascia to the mediastinum [4]. The dissemination of the DNI to the mediastinum can be facilitated by gravity, the pressure of the abscess, and negative thoracic pressure during respiration [41]. The symptoms of descending mediastinitis include odynodysphagia, dyspnea, respiratory distress, chest discomfort, and fever, even though the patients would not necessarily present these clinical manifestations [42]. This infectious illness most often occurs in middle-aged males, especially those with damaged immune function as well as nutritional insufficiency. Descending mediastinitis has a serious course, with the possibility of sepsis and septic shock, multiple organ failure, and even a high mortality rate [43]. A head and neck as well as a thoracic CT scan are the standard imaging tools to diagnose the disorder [44]. Pathogen culture often shows aerobic, anaerobic, or even mixed microorganisms based on pharyngeal or odontogenic sources [45,46]. The prognostic factors of DNI and descending mediastinitis, such as patient clinical data as well as laboratory findings with regard to outcome, have been discussed differently [4,6]. The concurrent diagnosis of DNI and descending mediastinitis usually requires treatment from a multidisciplinary department containing otorhinolaryngological care, infection control, thoracic surgery, and an intensive care unit for life-sustaining management [47]. In addition to effective antibiotic treatment, respiratory security and timely drainage of the deep neck space and mediastinum are mandatory [48]. The placement of drainage tubes and appropriate irrigation to prevent the possible formation of recurrent abscesses or the collection of purulent discharge are also necessary [47]. The empirical broad-spectrum antibiotic should be adjusted based on the cultured pathogen reports afterward. Patients with stable conditions who no longer require intravenous antibiotics can be discharged.

AI is a major topic in recent years. In fact, AI has been developing since the 1950s. However, in the past five years, the technological breakthrough of AI in clinical practices has shown amazing achievements in image recognition and language recognition. Through AI, the machine has the ability to learn and solve problems; let the machine learn the way of thinking like human beings. All industries expect to use AI as a powerful tool to assist in judgment, improve production efficiency, and achieve industrial upgrading. With the rapid development of science and technology, human beings will use AI technology in various practical aspects more frequently, and some specific fields have repeatedly surpassed the limits of human capabilities. This trend has also extended to medicine, and the transformation into an intelligent hospital is an indicator of the efforts of various hospitals. In the future, AI will have the opportunity to redefine the meaning of work. In the face of the coming era of AI, humans should consciously learn relevant knowledge in order to cope with the changes and challenges brought about by emerging AI technologies.

AI models can recognize complex patterns in images, text, sounds, and other data to generate more accurate insights and predictions. The useful models mainly make predictions depending on preceding datasets [20,49]. Several algorithms can be used to provide real-world clinical recommendations [50,51,52,53,54]. However, the performance of the model is hindered by the small size and poor quality of the training datasets [55]. The current medical utilizations of AI models comprise cancer diagnosis, integration of genomic data, clinical trial design, readmission analysis, and appropriate antibiotic treatment for infectious diseases [11,56,57,58,59].

Medical images are typically interpreted by human experts, such as radiologists and physicians. However, with advancements in medical imaging, it is now possible to load medical images onto a computer and perform automated analysis. In addition, medical imaging techniques have enhanced the creation of large databases that can be analyzed with AI systems. Thus, AI is important for the management of a large data volume and medical imaging interpretation [60,61,62].

Generally, humans make logical judgments and executions according to personal experience and different environments. Humans have emotional and personal preferences that can easily lead to differences in judgment results, which would cause errors. However, if consistency in judgments is essential, the methods and tools need to be systematic. AI can use systematic tools and algorithms to achieve decision-making logically and accurately via language or image recognition. Furthermore, AI can overcome fatigue and distraction and replace outdated techniques with new diagnostic techniques [63]. With advancements in AI algorithms, it is easier for computer scientists and healthcare researchers to collaborate together [64,65]. AI is also widely used in the field of otolaryngology [66,67,68,69,70,71,72]. In this research, we used a designed model to predict the progression of DNI to descending mediastinitis. Although AI has technical difficulties, humans can understand the basic concepts and operations of AI in the correct way.

Machine learning was established to overcome expert systems [11]. A prediction model is set up by providing historical data from patients, and machine learning tools are used to fit a model to this historical data [73]. The machine learning was applied after the data were converted to numerical input [13]. This technology was utilized for decision-making in infection control, risk of dementia, and predicting bone metastases of hepatocellular carcinoma [11,12,74]. Future work is required to compare the performance of various machine learning methods for the prediction of complications of DNI.

Among AI methods, the NN network algorithm is particularly useful due to its simplicity and high precision [75]. The *k*-NN algorithm is one of the most useful machine learning algorithms [28,76,77,78,79,80]. It is an important method for use in nonparametric algorithms [81]. The *k*-NN algorithm assumes that classification is based on the similarity of instances with their nearest neighbors (Figure 3). Compared to other classifiers, the *k*-NN algorithm has numerous advantages, including high versatility [23,25,82,83]. In this research, we used *k*-NN to evaluate DNI progressing to descending mediastinitis because there was less possibility of an in-between or indeterminate situation clinically or radiologically. The setting of a *k* value is typically based on the principle of odd numbers. The 1-NN classifier is typically used as a standard because it provides a logical analysis for several classification situations [26]. The *k*-NN algorithm basically indicates the contribution of the data information to the classification [84]. *k*-NN and distance-based AI are less robust than tree-based AI algorithms such as XGboost. XGBoost is a powerful ensemble learning method. Ensemble learning methods can combine the predictions of many individual trained classifiers. For XGBoost, the ensemble classifiers are decision trees [85]. The tree models are generally better for small datasets. t-SNE is a clustering and visualization method, and it has become a common tool in several natural sciences [86]. PCA is also a machine learning method [87], and it’s most commonly applied as an unsupervised algorithm. Future studies should explore the additional applications of AI from laboratory and clinical perspectives.

Some authors found that patients ≥ 55 years old, CRP ≥ 30 mg/dL, and neutrophil to lymphocyte ratio ≥ 13 prior to DNI management were analytic predictors of progression to descending mediastinitis. In this study, we applied the most significant variables to the training model [88,89,90]. The factors that lead to the high risk of progression of DNI to descending mediastinitis were selected. WBC count and CRP level are indicators of inflammation; patients with higher inflammatory markers have a higher possibility of infection progression. Some authors even found that increased CRP levels were a negative factor for the survival of patients with surgical treatment for descending mediastinitis in the postoperative period [43]. DM is reported to be an essential factor in determining the prognosis of patients with descending mediastinitis [7]. DM and hyperglycemic states also predispose to infection progression [29] and were included in the algorithm. Although the main route of dissemination of descending mediastinitis from DNI is the retropharyngeal space (around 70%) [44,91], the parapharyngeal and submandibular spaces can also spread infections to other spaces. Therefore, we included these three deep neck spaces in the model.

In fact, three different layers of deep cervical fascia make deep neck spaces as interconnected potential cavities, and these loose fascia layers cannot make the powerful barriers to resist the infection [92]. Involvement of multiple spaces (≥3) by DNI indicates severe infection [93] and was included in our model. Intubation and tracheostomy are the most common airway management strategies [94]. Thus, we included tracheostomy in the model. Furthermore, we considered the maximum diameter of the abscess and the shortest distance from the abscess to the level of the sternal notch in CT to be the most influential parameters for DNI progression to descending mediastinitis. In addition to representing the severity of the infection, the size of the abscess may also cause compression of the surrounding tissue or trachea. An abscess at the level of the sternal notch represents the possibility of DNI invading the mediastinum. Thus, these CT parameters were included in the study. Generally, a magnetic resonance scan is utilized as a secondary radiological tool to detect intracranial or spinal complications for DNIs [18].

Most DNI patients in our institution are not arranged for this imaging modality. As a result, this study did not include relevant data on magnetic resonance images of patients with DNIs.

### Study Limitations

Although we developed a preliminary prediction model for the prediction of DNI progression to descending mediastinitis, there were several limitations to the present study, including the use of retrospective data, reliance on self-reported medical history, subjective interpretation of CT, a small dataset, and data collection from a single institution. As a result, the model may only be applicable to populations similar to the population included in the present study. The distance between the abscess level and the level of the sternal notch in CT scans was measured manually for each person. The major issue in this article is that the dataset is highly imbalanced. Only minor populations with DNI progressing to mediastinitis are easily misleading. We did not handle the class imbalance problem in this study using, for example, the oversampling technique or SMOTE-like methods because they were proven to be dangerous for medical applications and failed to generate proper examples representing the minority class [95].

Because the applications of AI to DNI are still in the basic stage and the relevant references are relatively limited. Thus, there is no literature review section in this research. Our validation system was based on a 25/75% hold-out set for training and testing. It is suggested to repeat the experiment several times [96]. In this research, we did not perform an ablation study, which aims to investigate the performance of a model by removing certain portions of the algorithm. In addition, our model’s inability to achieve complete accuracy may be related to the limited variables used in the model. No validation group has been created to evaluate the overfitting issue. Furthermore, we did not use hyperparameter tuning, such as random search and parameters used for machine learning. This study lacked t-SNE or PCA, which could be used to reduce the model’s complexity or data dimensions to improve the model’s overall performance.

Future studies are needed to establish a comprehensive database that can be used to develop clinically relevant algorithms for predicting DNI progression to mediastinitis.

## 5. Conclusions

The aim of this research was to introduce the application of AI to clinical challenges in otorhinolaryngology. Our team developed a model to assess the progression of DNI to descending mediastinitis, depending on clinical and CT parameters. The model has prospective application, particularly for the prediction of risk for descending mediastinitis and the need for prompt treatment in patients with DNI. The model can assist clinicians in making decisions. However, the model is still in the basic stage. It is prudent for the reader to understand the potential benefits and limitations of these technologies via our research.

## Figures and Tables

**Table 1:** Clinical characteristics of 380 patients with deep neck infections.

Characteristics	*n* (%)
Age, years ± SD	51.12 ± 18.87
Gender	380 (100.0)
Male	255 (67.11)
Female	125 (32.89)
Chief complaint period, days ± SD	5.06 ± 4.47
WBC, µL ± SD	14,908.41 ± 5753.66
CRP, mg/L ± SD	154.68 ± 99.54
Blood sugar, mg/dL ± SD	142.56 ± 73.42
Diabetes mellitus	142 (37.36)
Parapharyngeal space involved	182 (47.89)
Submandibular space involved	167 (43.94)
Retropharyngeal space involved	100 (26.31)
Deep neck infection multiple spaces involved, ≥3	124 (32.63)
Tracheostomy performance	46 (12.11)
Maximum diameter of abscess, cm ± SD	6.23 ± 2.96
Nearest distance from abscess to level of sternum notch, cm ± SD	6.11 ± 3.92
Progression to mediastinitis	30 (7.89)

*n*, numbers; SD, standard deviation; WBC, white blood cell (normal range: 3500–11,000/µL); CRP, C-reactive protein (normal range < 5 mg/L); Blood sugar (normal range: 70–100 mg/dL); Maximum diameter of the abscess and the nearest distance from the abscess to the level of the sternum notch were evaluated in a CT scan.

**Table 2:** Comparison of clinical and imaging parameters between the training and test groups.

Characteristics	Training Group; *n* (%)	Testing Group; *n* (%)	*p*-Value
Age, years ± SD	50.75 ± 18.71	52.26 ± 19.38	0.521
Gender	285 (100.0)	95 (100.0)	
Male	195 (68.42)	60 (63.15)	0.378
Female	90 (31.58)	35 (36.85)	
Chief complaint period, days ± SD	5.33 ± 4.89	4.25 ± 2.71	0.213
WBC, µL ± SD	14,622.45 ± 5695.52	15,766.31 ± 5871.63	0.090
CRP, mg/L ± SD	150.97 ± 98.72	165.79 ± 101.66	0.191
Blood sugar, mg/dL ± SD	140.91 ± 72.55	147.51 ± 76.15	0.090
Diabetes mellitus			0.806
Yes	108 (37.89)	34 (35.78)	
No	177 (62.11)	61 (64.21)	
Parapharyngeal space involved	137 (48.07)	45 (47.36)	1.000
Submandibular space involved	119 (41.75)	48 (50.52)	0.152
Retropharyngeal space involved	68 (23.85)	32 (33.68)	0.079
Deep neck infection multiple spaces involved, ≥3	89 (31.22)	35 (36.84)	0.315
Tracheostomy performance			0.588
Yes	33 (11.57)	13 (13.68)	
No	252 (88.43)	82 (86.32)	
Maximum diameter of abscess, cm ± SD	6.08 ± 2.92	6.66 ± 3.04	0.072
Nearest distance from abscess to level of sternum notch, cm ± SD	6.26 ± 3.74	5.69 ± 4.41	0.210
Progression to mediastinitis			0.075
Yes	18 (6.31)	12 (12.63)	
No	267 (93.69)	83 (87.36)	

*n*, numbers; SD, standard deviation; WBC, white blood cell (normal range: 3500–11,000/µL); CRP, C-reactive protein (normal range < 5 mg/L); Sugar (normal range: 70–100 mg/dL); Maximum diameter of the abscess and the nearest distance from the abscess to the level of the sternum notch were evaluated in a CT scan.

## Data Availability

All data generated or analyzed during this study are included in this published article. The data are available on request.
